# Can we implement midwifery-led continuity of care in Ethiopia? Maternal health leaders and midwives perspective: a qualitative study

**DOI:** 10.1186/s12884-025-08319-z

**Published:** 2025-11-18

**Authors:** Yohannes Fikadu Geda, Ayenew Mose, Tamirat Melis Berhe, Fantaye Chemir, Keyredin Nuriye Metebo, Samuel Ejeta Chibsa, Seid Jemal Mohammed, Solomon Shitu Ayen, Molalegn Mesele Gesese, Ayele Sahile Abdo

**Affiliations:** 1 Department of Midwifery, College of Medicine and Health Science, Wolkite University, Wolkite, Ethiopia; 2 The Ritchie Centre, Hudson Institute of Medical Research, Clayton, Australia; 3 Department of Obstetrics and Gynaecology, Monash University, Clayton, Australia; 4 Department of Public Health, College of Medicine and Health Science, Wolkite University, Wolkite, Ethiopia; 5 Department of Midwifery, College of Medicine and Health Science, Mattu University, Mattu, Ethiopia; 6 Department of Public Health, College of Medicine and Health Science, Wolaita Sodo University, Wolaita Sodo, Ethiopia

**Keywords:** Midwifery-led, Continuity of care, Ethiopia, Maternal health, Perspectives

## Abstract

**Introduction:**

Recognition of the importance of midwifery-led continuity of care (MLCC) in promoting positive maternal and neonatal outcomes is remarkably increasing. Despite its potential benefits, the implementation of this model may face challenges, and understanding health professionals’ perspectives is crucial for successful integration into existing healthcare systems. Furthermore little is known about feasibility of implementation of MLCC model in Ethiopia. Therefor this qualitative study aims to explore the perspectives of maternal health leaders and midwives regarding the implementation of midwifery-led continuity of care.

**Methods and materials:**

A qualitative study was conducted in Guraghe zone health facilities, Central Ethiopia, from May 1st to 15th, 2022. Three focused group discussions among midwives and twenty in-depth interviews were conducted among either maternity ward head and/or chief executive directors/medical directors who were selected using a purposive sampling method. Data were first transcribed and then translated from local language to English. Thematic analysis was applied using open code software to identify recurring themes and patterns in the data, ensuring a comprehensive understanding of the professionals’ perspectives.

**Results:**

The findings reveal a spectrum of perspectives among maternal health leaders and midwives regarding MLCC. Positive themes include improved patient satisfaction, enhanced personalized care, and potential quality care. Conversely, challenges such as resistance to change, concerns about coordination, and the need for interdisciplinary collaboration were identified. The result favors implementing this model and underscores the importance of addressing issues for successful integration.

**Conclusion:**

Maternal health leaders and midwives acknowledge the potential benefits of MLCC model implementation besides recognizing the need for careful consideration of challenges in its implementation. During implementation by health facilities targeted training programs, inter-professional education, and strategic policy measures to facilitate a smooth transition are recommended.

## Introduction

MLCC is an innovative model of maternity care characterized by the consistent presence and guidance of a dedicated midwife throughout the entire childbirth continuum, encompassing prenatal, intra-partum, and postnatal periods [1, 2]. This approach emphasizes a woman-centered and holistic perspective, tailoring care to the unique needs of the expectant mother [3]. A midwife or small group of midwife assumes a central role in providing continuous support, fostering a collaborative and trusting relationship with the pregnant woman [4].

The model aims not only to address clinical aspects of care but also to integrate emotional, social, and cultural considerations, ensuring a comprehensive and personalized experience for the mother [5, 6]. This approach is designed to empower women and enhance overall well-being through a sustained and supportive partnership with a skilled midwife [7].

Despite improvements in service coverage, maternal mortality continues to pose a major public health challenge in Ethiopia [8]. In this context, the study explores the potential of midwifery-led continuity of care, an innovative approach characterized by a consistent midwife providing care throughout the childbirth continuum, encompassing pregnancy, labor, and the postpartum period [7].

The urgency of this research stems from the need to transform maternal healthcare delivery in Ethiopia [9]. Midwives, as skilled and empathetic healthcare providers, play a central role in promoting safe pregnancies and positive birth experiences [10]. This strategy aims to provide amplified insights into the practicality, acceptance, and potential barriers to the implementation of midwifery-led continuity of care [11].

Maternal health remains a critical global health concern, particularly in low-resource settings, where challenges persist in ensuring safe and effective maternity care [12]. Efforts to improve maternal health and reduce maternal mortality remain essential worldwide [13]. Despite reports of universal access to maternal and newborn health services in Ethiopia, low utilization persists, resulting in preventable deaths potentially linked to substandard care [14].

The presence and expertise of midwives significantly reduce maternal mortality rates, which could avert 41% of maternal deaths, 39% of neonatal deaths, and 26% of stillbirths, potentially saving around 2.2 million lives annually by 2035 [15]. The World Health Organization highlights midwifery care throughout childbirth can save women’s and newborns’ lives, with significant challenges and opportunities for improvement in low to lower-middle-income countries [12].

In Ethiopia, obstetric and maternity care is delivered by a variety of healthcare professionals, such as midwives, nurses, health officers and others, with variations in the quality of maternal and newborn health care, as indicated by a study reporting an average quality of 62% for the input component, 43% for the process component, and 48% for the output component among health facilities [14]. Basic Emergency Obstetric and Newborn Care training, on-job refresher training, and promotion of the midwifery profession are recommended strategies for enhancing maternity care tool utilization and overall maternity care [16]. However, the feasibility of introducing MLCC within the Ethiopian health system has not been adequately explored.

Previous studies in Ethiopia have largely focused on service utilization and outcomes of midwifery interventions, but little is known about the perspectives of midwives and maternal health leaders on the opportunities and barriers to implementing MLCC. This knowledge gap is particularly important as Ethiopia considers adopting MLCC as a national strategy.

This study therefore explores the views of midwives and maternal health leaders on the feasibility of MLCC in Ethiopia. By documenting their perspectives, the study contributes evidence to inform national policy debates and adds to the global literature on midwifery‑led models of care in low‑resource settings.

## Methods and materials

### Study design and period

This research employed a qualitative exploratory study design, utilizing semi-structured interviews and focus group discussions to gather in-depth insights into the perspectives of maternal health leaders, maternity ward heads and midwives regarding the feasibility and implementation of midwifery-led continuity of care in Guraghe zone, Central Ethiopia, from May 1 st to 15th, 2022.

### Study setting and participants

The study was conducted in Guraghe Zone, which comprises both urban and rural areas, incorporating healthcare facilities such as hospitals and health centers. Hospitals and health centers were purposively chosen to capture a comprehensive understanding of the challenges and opportunities for implementing midwifery-led continuity of care.

Healthcare administrators and maternity ward heads in the hospitals within Guraghe Zone, actively participated in the study. Additionally, experienced midwives, practicing professionals with a substantial background in providing maternal care services, were included as participants.

Maternal health leaders in this study included senior midwives, ward heads, and facility administrators. Obstetricians and members of the Ethiopian Society of Obstetricians and Gynecologists were not included, nor were national‑level policymakers. The perspectives of regional maternal health managers were captured where available.

### Sample size and sampling technique

About twenty health care leaders, heads of maternity wards, and senior midwives participated in the in-depth interview, enabling rich perspective exploration and data saturation. Three Focused Group Discussions (FGDs) with 6, 6, and 7 midwives were conducted to explore the group view as well as their perception and experience in feasibility of midwifery-led continuity care.

The study included the principal leader of the hospital, either the chief executive director or medical director, based on the hospital’s organizational structure, in addition with maternity ward heads. Purposive sampling was employed to ensure representation of diverse perspectives of midwives. Selection criteria included participants with extensive experience in maternal health and those directly involved in decision-making processes.

### Data collection tools

Tailored comprehensive interview guides were designed for both maternal health leaders and midwives, ensuring thorough coverage of relevant topics. To capture the richness of the discussions, high-quality audio-visual recording equipment was employed for both interviews and FGDs. This meticulous approach in the development of interview guides and the utilization of advanced audio-visual equipment aimed to enhance the depth and accuracy of data collection throughout the research process.

### Reporting framework and trustworthiness

This study was reported in accordance with the Consolidated Criteria for Reporting Qualitative Research (COREQ) checklist to ensure transparency and rigor.

Multiple strategies were employed to enhance trustworthiness. Credibility was supported through member checking and triangulation of data sources (midwives, ward heads, administrators). Dependability was strengthened through a detailed audit trail documenting coding and analytic decisions. Confirmability was strengthened through peer debriefing among the research team. Transferability was addressed by providing detailed descriptions of the study context and participant characteristics.

### In-depth interviews

The research employed a comprehensive interview guide, developed to cover topics including perceptions of midwifery-led continuity of care, potential benefits, challenges, and strategies for implementation. During face-to-face semi-structured interviews, participants had the opportunity to express their opinions freely. To ensure accuracy during subsequent transcription, all interviews were audio-recorded. The interviews took 30–45 min and it was conducted in a place that was convenient for participants.

### Focus group discussions (FGDs)

During the research process, a total of three FGDs were conducted among midwives to foster group dynamics and extract shared perspectives. These FGDs were systematically categorized into specific themes, including discussions on perceived barriers to implementation, collaborative strategies, and potential policy implications. Approximately 6–7 midwives actively participated in each FGD, ensuring a diverse and representative cross-section of perspectives within the midwifery community. To capture the richness of the discussions, including non-verbal cues and group interactions, the FGDs were recorded using audio-visual techniques. This comprehensive approach aimed to provide an accurate understanding of the midwives’ perspectives, facilitating a more in-depth exploration of the topics under consideration. The FGDs were conducted in a calm room with minimum sound disturbance and lasted 50 min to 1 h.

### Data management and quality control

A robust data management and validation process was implemented throughout the study. Transcripts, recordings, and any identifiable information were securely stored on password-protected devices to maintain confidentiality. Regular data backups were conducted to prevent potential loss. To enhance data validation, participants were offered the opportunity for member checking, allowing them to review and validate their transcripts. Peer debriefing was ensured through regular discussions among the research team, promoting reflexivity and validating the findings. Additionally, a triangulation approach was employed, utilizing multiple data sources and methods to ensure the study’s reliability and validity. This comprehensive approach aimed to uphold the integrity and trustworthiness of the research data.

Following data collection, a meticulous transcription process was undertaken for both recorded in-depth interviews and FGDs, ensuring a verbatim representation and preserving the authenticity of participants’ responses. Subsequently, two independent researchers conducted the coding of transcripts using a thematic coding framework, introducing a structured approach to analyzing the gathered data. To enhance the reliability of the coding process, regular meetings were conducted, fostering inter-coder reliability through discussions aimed at ensuring consistency and resolving any discrepancies in the coding interpretations. This rigorous methodology was employed to maintain accuracy and reliability throughout the analysis phase of the research.

### Data analysis

QDA Miner Lite software version 3.0.2 was used for qualitative data analysis and coding. The data analysis for this research employed a multifaceted approach to derive meaningful insights from the qualitative data. The results of the thematic analysis exposed participants’ varied points of view, which ranged from cautious optimism to passionate endorsement. However, major themes included the importance of individualized care, increased patient satisfaction, and possible gains in the overall efficiency of the health system. Moreover the study employed comparative analysis to examine the differences in replies from various participant groups, such as midwives, maternity ward heads and healthcare administrators. The analysis revealed both common and unique perspectives, issues, and suggested strategies. Content analysis systematically examined participant responses, identifying key themes related to potential benefits, challenges, and strategies for MLCC implementation. Pattern recognition techniques identified recurring themes, and the constant comparative method facilitated ongoing refinement of codes and themes. Contextual analysis explored how unique contextual factors influenced participant perspectives on MLCC.

## Results

### Study participants and engagement

There were 39 participants in total for this study, and 100% of them responded. During the study period, a total of 20 in-depth interviews were conducted, involving active participation from diverse stakeholders in the Guraghe Zone. Participants included healthcare administrators and maternity ward heads. In addition, 19 experienced midwives actively engaged in both semi-structured interviews and focus group discussions, contributing to a comprehensive and varied spectrum of perspectives. Total number of participant in this study was 39 with 100% of response rate.

A Maternity Ward Head emphasized the study’s significance, stating, *“This study is crucial for understanding how midwifery-led care could impact our maternal health services. It’s an opportunity to enhance our practices based on diverse perspectives.”*

Another participant, identified as a Midwife, shared their enthusiasm, stating, “*As a midwife*,* I’m excited to be part of this study. It’s a chance for us to contribute our insights and shape the future of maternal healthcare in Guraghe Zone.*” These sentiments underscore the collaborative nature of the study, emphasizing the importance of incorporating varied professional viewpoints into the research process.

### Themes emerged from in-depth interviews

#### Professional identity and autonomy

Participants emphasized that MLCC could strengthen midwives’ professional identity and autonomy. One midwife explained, “*If we are allowed to follow women throughout*,* we can show our real capacity.*” This theme reflects a desire for recognition and scope of practice expansion.

#### Perceptions of midwifery-led continuity of care

Participants articulated nuanced perspectives, ranging from enthusiastic endorsement to cautious optimism. Some expressed the belief that midwifery-led continuity of care could significantly enhance maternal health outcomes, while others highlighted the need for careful implementation considering contextual factors.

A Maternity Ward Head stated, “*I see potential in midwifery-led continuity of care. It aligns with our goal of providing personalized care. However*,* we must tread carefully and consider the unique challenges our healthcare system faces.”*

A Midwife added, *“I’m optimistic about midwifery-led care. It’s about tailoring care to the individual*,* which is fundamental. But we need to be mindful of how it fits into our existing practices.”*

#### Potential benefits

Participants identified a multitude of potential benefits, including increased maternal satisfaction, improved birth outcomes, and heightened continuity of care.

A Health Facility Administrator emphasized, *“Increased maternal satisfaction is a key benefit we hope to achieve. Satisfied mothers are more likely to engage in continued care and recommend our services.”*

A Midwife contributed, “*The potential for stronger patient-midwife relationships is immense. It could positively impact birth outcomes and overall patient experience.”*

#### Challenges

Participants candidly discussed various challenges, such as potential resistance to change, resource constraints, and the need for comprehensive training programs. Consistent challenges included concerns about the integration of midwifery-led care into existing healthcare structures, addressing resistance from other healthcare professionals, and ensuring sustainable funding for the initiative. Additionally, the hospitals need to employ more midwives than they have at this moment.

A Maternity Ward Head expressed concern, *“Change is often met with resistance. We need strategies to address this*,* especially from other healthcare professionals who might not fully understand the benefits.”*

A Midwife acknowledged, *“Resource constraints are a reality. Training programs need to be comprehensive; ensuring everyone involved is equipped with the necessary skills for successful implementation.”*

#### Strategies for implementation

Participants proposed an array of strategies, emphasizing the importance of collaborative decision-making, targeted training programs, and robust communication channels. Key strategies included community awareness campaigns, interdisciplinary collaboration, and the development of clear guidelines for midwifery-led care implementation.

A Health Facility Administrator suggested, “*Collaborative decision-making is crucial. We need buy-in from all stakeholders to ensure successful implementation.”*

A Maternity Ward Head added, *“Clear guidelines and communication channels are essential. It’s not just about implementation; it’s about maintaining the momentum for the long term.”*

### Themes identified from FGDs

#### Systematic barriers

Barriers included staff shortages, lack of supportive supervision, and resistance from other cadres. As one administrator noted, “*Without clear policy direction*,* it will be difficult to reorganize services.*” These findings highlight structural rather than individual challenges.

#### Collaborative strategies

Midwives discussed collaborative strategies, highlighting the importance of fostering positive relationships with other healthcare professionals, promoting interdisciplinary training programs, and establishing forums for open communication. Commonly suggested collaborative approaches were the development of shared care protocols, joint educational initiatives, and regular forums for healthcare professionals to discuss and address challenges collectively.

A Midwife emphasized, *“Positive relationships with other healthcare professionals are vital. We need joint training initiatives and regular forums for effective collaboration.”*

Another Midwife added, “*Shared care protocols and open communication channels can bridge the gap between different healthcare professionals and enhance the overall quality of care*.”

#### Opportunities for policy alignment

Several leaders linked MLCC to Ethiopia’s broader maternal health strategies, suggesting that piloting MLCC could inform national scale‑up.

## Discussion

This study provides timely insights into the feasibility of MLCC in Ethiopia. Participants recognized the potential of MLCC to improve continuity, quality, and women’s experiences of care, but also identified systemic barriers such as workforce shortages, unclear role definitions, and resistance from other professional groups. The results of this study offer significant insights into MLCC, unveiling both consistencies and distinctive considerations within the healthcare landscape of the Guraghe Zone. The evident robust engagement of participants aligns with research emphasizing the critical role of diverse stakeholder representation in shaping midwifery-led healthcare models. The study’s findings contribute to the growing understanding of MLCC by highlighting the importance of varied participant profiles, reflecting the dynamic interplay of the implementation dynamics of MLCC.

This study looks at what people think about and revealed that healthcare system work better shows a positive outlook, thinking that MLCC could make big improvements. This is similar with a study conducted in Northern Ethiopia [1]. The different views from both studies show that healthcare workers have different thoughts about MLCC, but there are some good things that everyone seems to agree on. In the study from Northern Ethiopia, they liked the idea of giving more personalized care, making patients happier, and making the healthcare system work better. These positive points are like common ground that people globally can agree on. By connecting with the positive findings in the Northern Ethiopia study, this research not only helps us understand more about MLCC but also shows that it could bring good changes to healthcare in different places.

In examining the findings of this study, it becomes evident that the challenges identified, encompassing potential resistance to change and concerns about integration complexities, resonate with broader themes found in existing literature on the implementation of novel healthcare models. This alignment is particularly noticeable when considering studies conducted in low- and middle-income countries, where a prevailing theme is the widespread resistance and challenges encountered in the adoption of innovative care approaches [17, 18]. The recurrent emphasis on the necessity for comprehensive training programs, as identified in our study, echoes a consistent thread in the existing body of literature, underscoring the critical role of ongoing education in facilitating successful transitions to new care models [19, 20]. The recognition of these challenges not only contextualizes the current study within a larger framework but also emphasizes the shared nature of obstacles faced in the global landscape of healthcare innovation and transformation.

The strategies articulated by participants in our study resonate closely with the recommendations drawn from successful implementations of MLCC as outlined in studies focusing on leadership and management within midwifery-led care models [20]. The emphasis on collaborative decision-making, targeted training programs, and the establishment of robust communication channels aligns seamlessly with recognized best practices in implementing patient-centered care models. This alignment across various studies not only reinforces the importance of these strategies but also enhances the evidence supporting their effectiveness in overcoming challenges commonly associated with healthcare model transitions [21–24]. The consistent endorsement of these approaches across diverse contexts strengthens the argument for their applicability and success in the broader landscape of healthcare innovation and patient-centric care delivery.

Insights from focus group discussions (FGDs) in our study align with studies on improving outcome of maternal and neonatal health [18, 25, 26], these studies say it’s important to clear up misunderstandings and create a supportive work environment for midwives. The perceived barriers and collaborative strategies identified in our FGDs mirror findings in a study with the title barriers and facilitators to the implementation of midwife-led care [17]. This shows that it’s a common need everywhere to deal with resistance and work together across different parts of healthcare. It’s like a reminder that making sure everyone understands and works together is important for making care better for moms and babies around the world.

The conversation about possible policy implications in our study aligns with the larger body of literature that promotes policy reforms to back innovative care models. A study titled “Transforming Maternity Care Globally” [4] highlights the importance of policy changes in making it easier to include midwifery-led care and making sure midwives are acknowledged in policy-making discussions. This connection underscores the widespread call for policy adjustments to support and integrate new and effective care models, emphasizing the critical role of midwifery and advocating for the inclusion of midwives in policy decisions on a global scale.

Our findings align with international evidence showing that midwives often face constraints in practicing to their full scope, including lack of autonomy and hierarchical resistance. Studies from Bangladesh and other low‑ and middle‑income countries demonstrate that supportive environments, mentoring, and clear policy frameworks are essential for enabling midwives to deliver high‑quality care [27, 28].

### Policy and practice implications

For Ethiopia, introducing MLCC will require:

- Clear policy directives defining midwives’ scope of practice.
- Workforce planning to ensure adequate numbers of midwives.
- Supportive supervision and inter‑professional collaboration.
- Pilot programs to generate local evidence on implementation.

### Limitations

This study was conducted in Guraghe Zone and may not reflect perspectives from other regions or national‑level stakeholders. As with all qualitative research, the findings are not statistically generalizable; however, they offer valuable in‑depth insights into feasibility and stakeholder perspectives. Perspectives of obstetricians and national policymakers were not included, which may limit the comprehensiveness of stakeholder views.

## Conclusion and recommendation

Thematic analysis of the collected data illuminated important spectrum of viewpoints among maternal health leaders and midwives with regard to MLCC. Positive themes surfaced, encompassing anticipated enhancements in patient satisfaction, the provision of more personalized care, and the potential for elevated overall care quality. However, the study discover challenges, including entrenched resistance to change, concerns about coordination complexities, and the essential need for robust interdisciplinary collaboration.

The findings elucidates that maternal health leaders and midwives in Guraghe Zone recognize the promising benefits of MLCC but advocate for a thoughtful approach to mitigate potential challenges. While both maternal health leaders and midwives acknowledged the potential benefits of MLCC, they concurrently recognized the imperative of meticulous consideration and strategic planning to navigate the anticipated challenges during the implementation phase.

Comprehensive training programs should be instituted to enhance understanding and foster a shared vision of MLCC benefits. The establishment of platforms for consistent communication and change management techniques is crucial to overcome ingrained resistance, as interdisciplinary collaboration is essential. Pilot programs, feasibility studies, and policy advocacy are essential steps, ensuring a phased and evidence-based approach to MLCC integration. Continuous evaluation, community awareness campaigns, strategic resource allocation, and active participation in research initiatives will further optimize the success of MLCC, contributing to enhanced maternal care quality and patient satisfaction in Guraghe Zone.

## Data Availability

The data that support the findings of this study are available but restrictions apply to the availability of these data, which were used under license for the current study, and so are not publicly available. Data are however available from the first author upon reasonable request.
